# Environmental Drivers of Differences in Microbial Community Structure in Crude Oil Reservoirs across a Methanogenic Gradient

**DOI:** 10.3389/fmicb.2016.01535

**Published:** 2016-09-28

**Authors:** Jenna L. Shelton, Denise M. Akob, Jennifer C. McIntosh, Noah Fierer, John R. Spear, Peter D. Warwick, John E. McCray

**Affiliations:** ^1^Eastern Energy Resources Science Center, U.S. Geological SurveyReston, VA, USA; ^2^National Research Program-Eastern Branch, U.S. Geological SurveyReston, VA, USA; ^3^Department of Hydrology and Atmospheric Sciences, University of ArizonaTucson, AZ, USA; ^4^Department of Ecology and Evolutionary Biology, University of ColoradoBoulder, CO, USA; ^5^Cooperative Institute for Research in Environmental Science, University of ColoradoBoulder, CO, USA; ^6^Department of Civil and Environmental Engineering, Colorado School of MinesGolden, CO, USA; ^7^Hydrologic Science and Engineering Program, Colorado School of MinesGolden, CO, USA

**Keywords:** oil field, microbial ecology, methane, hydrogeochemical tracers, methanogenic crude oil biodegradation, Gulf Coast Basin

## Abstract

Stimulating *in situ* microbial communities in oil reservoirs to produce natural gas is a potentially viable strategy for recovering additional fossil fuel resources following traditional recovery operations. Little is known about what geochemical parameters drive microbial population dynamics in biodegraded, methanogenic oil reservoirs. We investigated if microbial community structure was significantly impacted by the extent of crude oil biodegradation, extent of biogenic methane production, and formation water chemistry. Twenty-two oil production wells from north central Louisiana, USA, were sampled for analysis of microbial community structure and fluid geochemistry. Archaea were the dominant microbial community in the majority of the wells sampled. Methanogens, including hydrogenotrophic and methylotrophic organisms, were numerically dominant in every well, accounting for, on average, over 98% of the total Archaea present. The dominant Bacteria groups were *Pseudomonas, Acinetobacter*, Enterobacteriaceae, and Clostridiales, which have also been identified in other microbially-altered oil reservoirs. Comparing microbial community structure to fluid (gas, water, and oil) geochemistry revealed that the relative extent of biodegradation, salinity, and spatial location were the major drivers of microbial diversity. Archaeal relative abundance was independent of the extent of methanogenesis, but closely correlated to the extent of crude oil biodegradation; therefore, microbial community structure is likely not a good sole predictor of methanogenic activity, but may predict the extent of crude oil biodegradation. However, when the shallow, highly biodegraded, low salinity wells were excluded from the statistical analysis, no environmental parameters could explain the differences in microbial community structure. This suggests that the microbial community structure of the 5 shallow, up-dip wells was different than the 17 deeper, down-dip wells. Also, the 17 down-dip wells had statistically similar microbial communities despite significant changes in environmental parameters between oil fields. Together, this implies that no single microbial population is a reliable indicator of a reservoir's ability to degrade crude oil to methane, and that geochemistry may be a more important indicator for selecting a reservoir suitable for microbial enhancement of natural gas generation.

## Introduction

Increasing demands for energy combined with diminishing economically accessible fossil fuel reserves will require novel energy-efficient technologies for hydrocarbon production. There are large, historically non-viable estimated global reserves of both heavily biodegraded (i.e., microbially altered) crude oil and residual crude oil (crude oil that remains *in situ* following traditional extraction mechanisms). However, these reservoirs are increasingly being considered a potentially viable contribution to the energy mix as lighter, easier-to-produce crude oils diminish (Hein et al., 2013). One suggested mechanism to “produce” this difficult-to-obtain oil is through enhancing *in situ* microbial communities to metabolize the oil to natural gas via methanogenic crude oil biodegradation (Parkes, 1999; Gieg et al., 2008; Jones et al., 2008; Head et al., 2014; Meslè et al., 2015). However, significant technical barriers to commercial development remain, including identification and manipulation of the most important biogeochemical factors controlling bioconversion.

A consortium of microorganisms, most notably, methanogens and syntrophic bacteria, perform methanogenic crude oil biodegradation by breaking down crude oil into methanogenic substrates (e.g., formate, acetate, carbon dioxide), which are subsequently converted into natural gas. The process has been confirmed in laboratory studies where microbial communities collected from deep methanogenic oil reservoirs can consume crude oil and produce methane (e.g., Gieg et al., 2008; Berdugo-Clavijo et al., 2012; Berdugo-Clavijo and Gieg, 2014). In addition, subsurface communities capable of converting oil to natural gas have been described using a variety of sequencing techniques, but community structure (abundance and diversity) varied between studies (e.g., Dahle et al., 2008; Pham et al., 2009; Shartau et al., 2010; Yamane et al., 2011; Kryachko et al., 2012; Li et al., 2012; Berdugo-Clavijo and Gieg, 2014; Meslè et al., 2015). Despite this knowledge, we are still limited in our understanding of how hydrochemistry and microbial populations may impact methanogenic crude oil biodegradation under fairly constant lithological conditions (e.g., Kirk et al., 2015). Most of the previous studies were performed on a small scale (i.e., a small number of samples from a hydrogeochemically similar setting), while the few large datasets rarely compare microbial communities across environmental gradients. In addition, it is difficult to analyze the impact of hydrogeochemical conditions across samples collected from varying lithologies, because lithology has a causative impact on hydrochemistry.

The objective of this study was to expand on current knowledge about the microbiology of methanogenic crude oil biodegradation by sampling biomass across a hydrogeochemical gradient within the same lithologic formation exhibiting varying extents of methanogenesis and crude oil biodegradation. To accomplish this objective, we characterized microbial communities from 22 different oil wells from 6 different oil fields across a transect; all wells produce from the Wilcox Group of north-central Louisiana, USA. Previous work (Warwick et al., 2008; McIntosh et al., 2010; Shelton et al., 2014, 2016) established the amount of crude oil biodegradation, relative amount of microbial methanogenesis, and the formation gas and water geochemistry across north-central Louisiana. The new microbial data, combined with previous analyses of fluid chemistry, enabled us to explore the environmental conditions and microbial populations most conducive to methanogenic crude oil biodegradation.

## Study site and methods

### Study site

**Figure 1:**
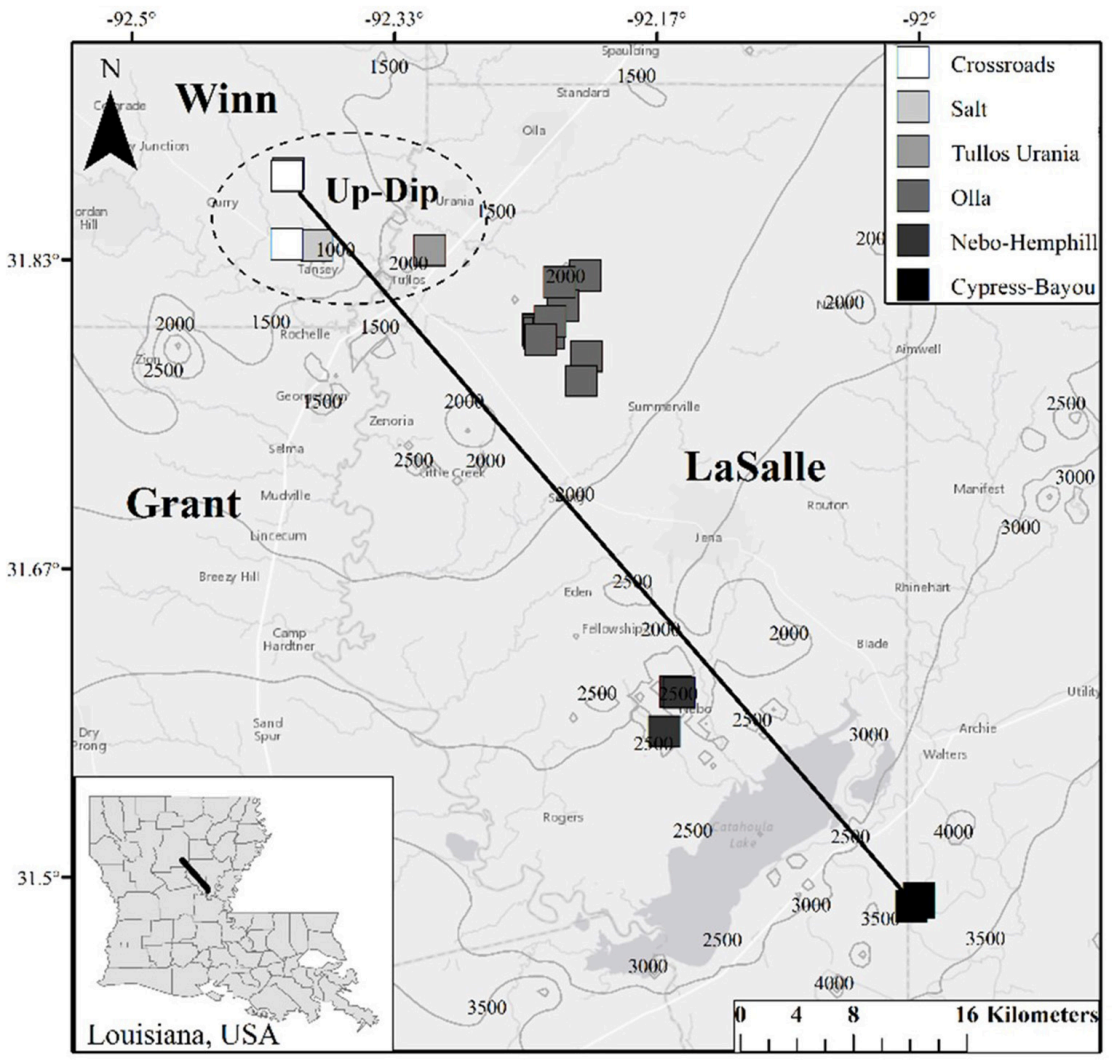
**Map of well locations sampled for this study**. Contours on the map indicate depth (in feet; 1 meter = 3.28084 feet) to the top of the Wilcox Group in the region, and Louisiana parishes (i.e., counties) are labeled. The dashed circle indicates the locations of the 5 up-dip wells referred to here; the remaining non-circled wells are referred to as the down-dip oil fields/wells within the text. The black line (i.e., the transect) is approximately 65 km long.

Twenty-two oil wells (Table S1), spanning 6 oil fields across a approximately 65 km transect producing from the Paleocene—Eocene Wilcox Group were sampled in 2014 (Figure 1). Biomass, produced water, and 4 oil samples were collected in this 2014 sampling campaign. These data were supplemented by produced gas and oil data collected from a 2011/2012 sampling campaign (Shelton et al., 2014, 2016). We justified the use of this complimentary, older dataset (oil and gas data from 2011/2012) due to slow rates of crude oil biodegradation and subsequent methanogenesis (e.g., Larter et al., 2003, 2006), and because chemical water analyses from this sampling campaign produced values similar to those from 2011/2012 (Shelton et al., 2014). The sampled wells spanned a wide range of geochemical parameters, including temperature, pH, and salinity (Table 1), and formation fluids with varying extents of both crude oil biodegradation and microbial methanogenesis (McIntosh et al., 2010; Shelton et al., 2014, 2016). One oil field sampled, the Olla Field, has been previously suggested to be a hot spot of microbial methanogenesis in the region (McIntosh et al., 2010), later attributed to ideal geochemical conditions for microbial methanogenesis (Shelton et al., 2014).

**Table 1:** **Major field and geochemical parameters from this study and others and associated bins for statistical analyses**.

**Well ID**	**Oil Field**	**Depth (m)**	**This Study**					**Previous Work**				
			**Temp. °C**	**pH**	**δ^13^C-DIC‰**	**Alk. meq/kg**	**Cl-mM**	δ^13^C-CO_2_^*^‰	δ^13^C-CH_4_^*^‰	**Extent of Meth.^^^**	**Extent of COB^^^**	**CH_4_ Prod.^^^ m^3^ CH_4_/day**
CR1	Crossroads	438.3	28.7	7.6	−2.2	13.4	567	n/a	n/a	Low	High	BDL
CR2	Crossroads	439.8	25.5	7.7	−2.0	13.2	559	n/a	n/a	Low	High	BDL
CR3	Crossroads	472.4	24.2	7.6	−2.0	13.2	564	n/a	n/a	Low	High	BDL
S3	Salt	433.7	28.4	7.5	−0.3	10.2	685	n/a	n/a	Low	High	BDL
TU1	Tullos Urania	393.2	32.0	7.2	2.9	7.4	776	n/a	n/a	Low	High	BDL
O3	Olla	828.4	39.8	7.1	16.9	37.7	1382	12.8	−52.3	High	Minimal	590,000
O4	Olla	850.4	41.7	6.7	15.7	40.8	1394	10.9	−45.8	Medium	Minimal	160,000
O5	Olla	833.3	38.0	7.0	22.8	34.8	1334	17.2	−45.3	Medium	Moderate	110,000
O6	Olla	853.7	43.2	6.7	21.8	45.6	1394	16.7	−46.3	High	Moderate	390,000
O7	Olla	850.4	41.8	7.4	21.5	46.2	1371	16.2	−46.9	High	Moderate	390,000
O8	Olla	856.5	40.3	7.1	23.6	52.2	1351	17.7	−49.7	High	Moderate	460,000
O18	Olla	833.9	38.4	6.9	21.2	44.1	1385	15.8	−47.6	Medium	Moderate	130,000
O26	Olla	679.4	33.7	6.9	23.4	38.2	1154	15.8	−48.3	High	Minimal	430,000
O27	Olla	666.9	33.6	7.1	23.6	51.8	1117	17.4	−45.3	High	Minimal	190,000
O31	Olla	1043.6	48.5	6.9	21.7	56.4	1678	n/a	n/a	High	n/a	n/a
O32	Olla	856.5	40.3	6.9	24.1	53.1	1385	n/a	n/a	High	n/a	n/a
NH1	Nebo-Hemphill	1208.2	44.7	6.9	8.6	5.8	1676	1.8	−69.0	Low	Moderate	n/a
NH2	Nebo-Hemphill	1062.5	45.0	6.9	16.0	10.3	1512	n/a	−48.9	Medium	n/a	n/a
NH3	Nebo-Hemphill	1211.9	38.0	6.8	8.3	6.7	1687	2.2	−56.4	Low	Minimal	25,000
CB1	Cypress-Bayou	1472.8	24.4	6.8	1.2	5.0	2020	−6.3	−62.7	Low	Minimal	BDL
CB3	Cypress-Bayou	1485.3	29.4	6.9	0.2	4.7	2056	−6.8	−63.2	Low	Minimal	BDL
CB4	Cypress-Bayou	1588.0	28.8	7.3	0.7	5.6	2000	n/a	n/a	Low	Minimal	BDL

* Shelton et al. (2014);

^ *Shelton et al. (2016)*.

*Fill color coordinates to the bin each sample value was placed in for statistical analysis. Red, Highest; Orange. High; Tan, Medium; Light Blue, Low; Blue, Lowest. Cells labeled “n/a” (white) were not binned*.

*Temp., Temperature; DIC, Dissolved Inorganic Carbon; Alk., Alkalinity; CO_2_, Carbon Dioxide; CH_4_, Methane Gas; Meth., Methanogenesis; COB, Crude Oil Biodegradation; BDL, Below Detection Limit; CH_4_ Prod., methane produced from the wellhead*.

*n/a Indicates that the sample(s) were not available for analysis*.

### Field and analytical methods

Formation oil, water, and gas were collected over two different sampling campaigns in August 2011, and July 2012, while biomass and formation water and oil were collected in August 2014 (Table S1). Due to the similarity of the produced water geochemistry collected in 2011/2012 compared to 2014, along with slow rate constants for crude oil biodegradation (e.g., Larter et al., 2006), major changes in methanogenic activity and crude oil composition were not expected over the sampled time scale (2–3 years). Each well is identified by oil field and a sample number (Table 1, Table S1). The up-dip, shallow (393–472 m) wells from the Crossroads, Salt, and Tullos Urania fields are identified as CR, S, or TU wells, respectively. The mid-depth (667–1044 m), centrally-located wells from the Olla and Nebo-Hemphill fields are identified as O or NH, respectively. The deep (1063–1588 m), down-dip wells from the Cypress-Bayou Field are identified as CB.

Formation water and oil were collected directly from the wellhead into a 5 gallon Nalgene carboy, where the temperature of the brine/oil mixture was taken while the mixture partitioned, using an Oakton Acorn Temp 6 Thermometer (Vernon Hills, Illinois USA). Once separated, the water was released from a spigot at the bottom of the carboy into sterile 60 mL BD Luer-Lok syringes, and filtered through attached 0.2 μm nylon syringe filters into 30 mL HDPE bottles. The HDPE bottles were pre-cleaned and the collected fluids were preserved according to associated analysis (e.g., Shelton et al., 2014), filled with no headspace, capped, put on ice, and shipped to either the University of Arizona or the U.S. Geological Survey (USGS) where they were kept at 4°C until analyzed.

Produced fluids were collected in 2 L pre-combusted, sterile glass bottles for filtering microbial biomass. Nalgene tubing was inserted into the bottle after the oil/brine mixture was allowed to partition, and the partitioned brine was filtered through sterile 0.22 μm Sterivex™ GP filter units (Millipore®, Billerica, MA USA) using a GeoPump™ (Geotech Environmental Equipment, Inc. Denver, CO). The volume of filtered water was recorded, and triplicate filters were taken for each well. Filters were immediately frozen on dry ice and shipped to Colorado School of Mines, where they were kept at −80°C until DNA extraction. Alkalinity was titrated within 8 h of sample collection following the protocol outlined in Gieskes and Rogers (1973), and pH was recorded in the field using a Thermo Scientific Orion pH electrode. The δ^13^C-DIC of produced water was measured at the University of Arizona's Environmental Isotope Laboratory using a ThermoQuest Finnigan Delta Plus XL continuous flow gas ratio mass spectrometer (precision at least ±0.3%0). Anions were measured using ion chromatography at the USGS Energy Resources Program Geochemistry Laboratory in Lakewood, Colorado.

### Microbial community analysis

DNA extractions, amplifications, and Illumina MiSeq 16S rRNA gene sequencing were performed at the University of Colorado Next Generation Sequencing Facility. DNA was extracted using a MO BIO PowerSoil® DNA Isolation Kit (Mo BIO Laboratories, Carlsbad, CA) following the manufacturer's protocol, with slight modifications for the Sterivex™ GP filter units: the Sterivex filter units were opened and slices of the filter were added directly to the bead tubes. Extracted DNA was amplified using the 515-F (5′-GTGCCAGCMGCCGCGGTAA-3′) and 806-R (5′-GGACTACHVGGGTWTCTAAT-3′) 16S rRNA gene primer pair (Fierer et al., 2012); the primers also included Illumina adapters and reverse primers (adapted with error-correcting 12-bp barcodes unique to each sample). PCR was performed with a GoTaq® Hot Start PCR Master Mix (Promega, Madison, WI USA) in a 25 μL reaction, with thermal cycling consisting of initial denaturation at 94°C for 3 min, followed by 35 cycles (45 s) of denaturation at 94°C, annealing at 50°C for 30 s, extension at 70°C for 90 s, and a final extension at 72°C for 10 min. Successful amplification was verified with gel electrophoresis (2% agarose gel), and the amplified DNA was sequenced using the Illumina MiSeq platform running 2 × 250 bp chemistry (Illumina, San Diego, CA USA).

Downstream processing was performed using the UPARSE pipeline (Edgar, 2013), where forward reads were demultiplexed using an in-house custom Python script (https://github.com/leffj/helper-code-for-uparse). Quality filtering was conducted using a maxee value of 0.5 (filtering out ca. 10% of the nucleotides). Prior to determining and assigning phylotypes, the sequences were dereplicated and singleton sequences were removed. Taxonomic units were mapped to the raw sequences at the 97% similarity threshold using the Greengenes 13_8 (http://greengenes.secondgenome.com) database. Any sequences that matched phylotypes representing chloroplasts or mitochondria were removed, along with sequences matching those found in the blank sample (i.e., sequencing contaminants). The samples were then rarefied randomly at 3700 sequences per sample using the biom package in R (McDonald et al., 2012; http://www.r-project.org). Sequence reads for each well were deposited to the National Center for Biotechnology Information Short Read Archive (SRA) under BioProject PRJNA310850 and BioSample accession numbers SAMN04457226-SAMN04457247.

### Microbial community statistics

The 22 sampled wells were binned according to each of the following environmental parameters in order to determine how changes in environmental parameters impacted microbial community structure: oil field and well depth; temperature, pH, chloride concentration, indicators of extent of methanogenesis (δ^13^C-DIC values, alkalinity, methane production volumes, δ^13^C-CO_2_ values, and δ^13^C-CH_4_ values), and extent of crude oil biodegradation (Table 1). Each sampled well was assigned to one of 3–5 categories—Lowest, Low, Medium, High, or Highest—for each environmental parameter analyzed for that sample. The thresholds for these bins were determined so that a relatively even number of samples (e.g., 5–6 samples in each of the 3–5 categories) were placed in each bin (Table 1). The binning process was aided by natural cut-offs for many of the environmental parameters (e.g., chloride concentrations increased from 776 to 1154 mM between two different oil fields). Extent of methanogenesis and extent of crude oil biodegradation rankings for each well were assigned using indicators of methanogenesis (e.g., δ^13^C-DIC values) and crude oil geochemistry (e.g., pristane/phytane values), respectively (see Shelton et al., 2016 for methodology). Any well with missing data for a specific geochemical analysis was omitted from any statistical analyses performed using that parameter. All statistical analyses were conducted using the vegan package (Oksanen et al., 2015) in R (http://www.r-project.org).

## Results and discussion

### Produced fluid geochemistry

Formation water chemistry was sampled in both 2011/2012 and for this study, but only the data for the current sampling campaign will be presented here. Formation water temperatures ranged from 22.4° to 48.5°C across the sampled transect, with the coldest water located in the Crossroads Field and the warmest water located in the Olla Field (Table 1). The pH ranged from 6.7 to 7.7, with the lowest pH found in the Olla Field and the highest pH found in the Crossroads Field. Alkalinity values ranged from 4.7 to 56.4 meq/kg, with the highest values (≥13.4 meq/kg) in the Olla Field. Chloride concentrations ranged from 558 to 2056 mM, generally increasing with increasing well depth along the transect. The δ^13^C-DIC values of produced water ranged from −2.2 to +24.1%0, with the highest values found in the Olla and Nebo-Hemphill fields. The δ^13^C-CO_2_ and δ^13^C-CH_4_ values were taken from Shelton et al. (2014), as gas samples were not collected for this study. Daily gas production volumes were supplied by XTO Energy (written communication), and were converted to daily methane production volumes (Table 1) using gas composition data presented in Shelton et al. (2014).

### Differences in microbial community composition across the transect

The total number of reads per well varied across the transect, with an average of 21,795 reads per well (range: 3925–66,965 reads, Table 2). The wells sampled for this study contained a total of 2668 unique operational taxonomic units (OTUs), with 4.8% of the total OTUs identified at the species level (128 OTUs out of 2668). On average, there were 250 OTUs detected per collected sample, with 236 different bacterial OTUs and 13 archaeal OTUs identified.

**Table 2:** **Basic Taxonomic data identified from formation water**.

**Well ID**	Number of Reads^*^	**Species Richness^^^**	**Pielou's Evenness**	**% Archaea**	**% Bacteria**	**Most Abundant OTU (Archaea)**	**Most Abundant Bacteria**
CR1	29,076	438	0.82	59.7	40.3	*Methanococcus* spp.	*Flexistipes* spp.
CR2	46,150	442	0.81	75.8	24.2	*Methanococcus* spp.	*Chromatiales*
CR3	66,965	655	0.71	52.1	47.9	*Methanococcus* spp.	*MSBL8*
S3	12,954	246	0.90	78.1	21.9	*Methanococcus* spp.	*Desulfuromonadales*
TU1	35,894	316	0.79	42.5	57.5	*Methanococcus* spp.	*Clostridiaceae*
O3	8557	268	0.84	26.9	73.1	*Methanothermococcus* spp.	*Alicyclobacillus* spp.
O4	5929	144	0.89	42.9	57.1	*Methanothermococcus* spp.	*Alicyclobacillus* spp.
O5	28,714	219	0.69	65.9	34.1	*Methanothermococcus* spp.	*Deferribacter* spp.
O6	45,552	138	0.82	72.2	27.8	*Methanohalophilus halophilus*	*Thermovirgaceae*
O7	13,711	120	0.80	40.4	59.6	*Methanothermococcus* spp.	*Acinetobacter lwoffii*
O8	10,475	335	0.88	38.3	61.7	*Methanothermococcus* spp.	*Alicyclobacillus* spp.
O18	13,167	270	0.83	53.8	46.2	*Methanothermococcus* spp.	*Alicyclobacillus* spp.
O26	17,624	197	0.77	55.6	44.3	*Methanothermococcus* spp.	*Thermovirgaceae*
O27	39,069	166	0.60	78.3	21.7	*Methanothermococcus* spp.	*BA201*
O31	3925	255	0.77	37.9	62.1	*Methanothermococcus* spp.	*Halanaerobium* spp.
O32	5510	229	0.888	8.3	91.7	*Methanothermococcus* spp.	*Alicyclobacillus* spp.
NH1	4858	200	0.82	29.9	70.1	*Methanothermococcus* spp.	*Chitinophagaceae*
NH2	18,430	173	0.82	50.0	50.0	*Methanolobus* spp.	*Thermovirgaceae*
NH3	17,603	117	0.84	86.7	13.3	*Methanothermococcus* spp.	*Alicyclobacillus* spp.
CB1	5966	126	0.84	51.0	49.0	*Methanothermococcus* spp.	*Methylobacterium komagate*
CB3	5131	166	0.87	11.0	89.0	*Methanothermococcus* spp.	*Plesiomonas shigelloides*
CB4	44,233	290	0.62	70.9	29.1	*Methanothermococcus* spp.	*Calanaerocella* spp.

* *Calculated prior to rarefaction*.

^ *Number of species identified after rarefaction at > 0% abundance*.

**Figure 2:**
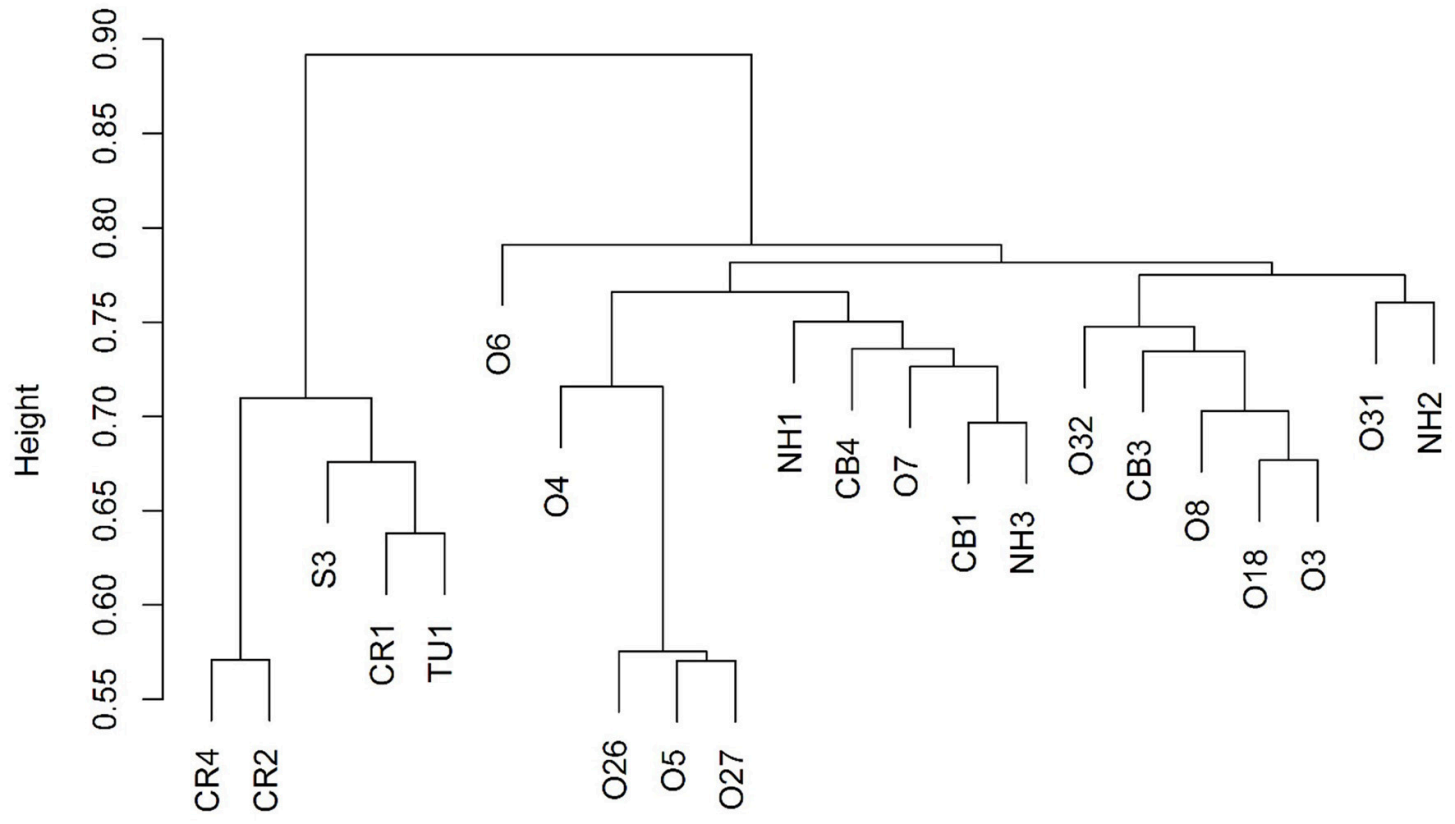
**Cluster dendrogram showing that the 5 up-dip wells (CR, S, and TU) cluster away from the remaining 17 down-dip wells**.

Archaea were observed at a higher average percent abundance than bacteria in most of the 22 sampled wells, ranging from 8.3 to 86.7% of total abundance (Figure 2, Table 2). A methanogen, regardless of the percent abundance of Archaea, was always the dominant OTU in each well, and methanogens, on average, represented 98.6% of the archaeal population (Figure S1). Bacteria were generally dominated by Proteobacteria (with *Alphaproteobacteria* or *Gammaproteobacteria* found at the highest abundance), Firmicutes, Bacteroidetes, and Actinobacteria (Figure S1). However, only 8 bacterial phyla were observed, on average, at greater than 1% abundance across the transect.

**Figure 3:**
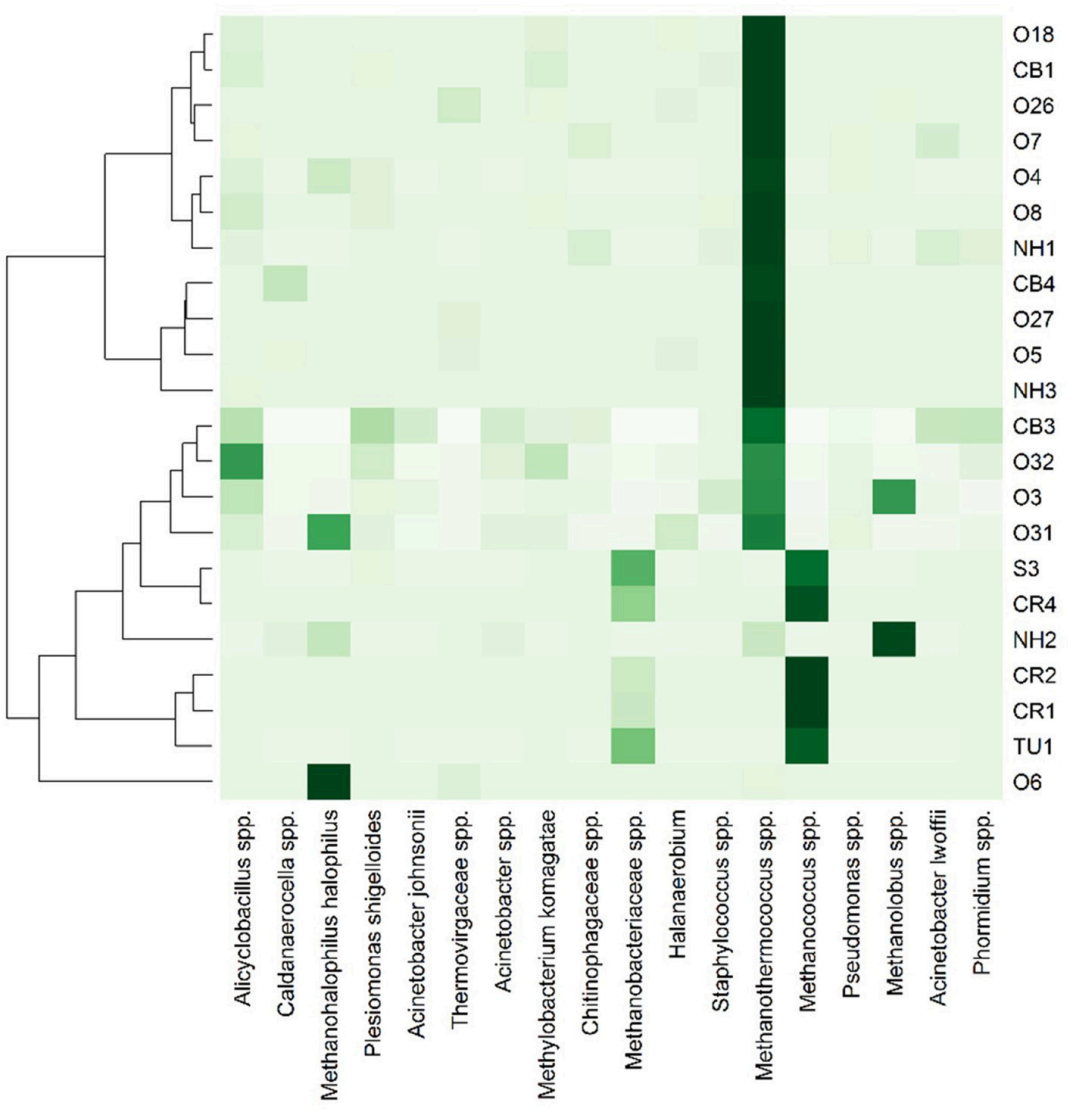
**Heat map representation of most abundant (i.e., >10% in at least one well) microbial taxa across the transect**. Darker colors indicate greater percent abundance than the lighter colors. The taxa found in greatest abundance across the transect are *Methanohalophilus halophilus, Methanothermococcus* spp., *Methanococcus* spp., and *Methanolobus* spp. 235 OTUs were identified at >1% abundance in at least one well sampled.

Only 18 different OTUs were present at greater than 10% abundance in any well sampled, 5 of which were methanogens (Figure 3; R commands: as.dendrogram; heatmap). *Methanococcus* spp. dominated the microbial structure of the 5 shallow, low salinity (up-dip) wells, which were also characterized by low temperatures and high extent of crude oil biodegradation. *Methanothermococcus* spp. was the most abundant microorganism in 15 of the wells (Figure 3), which were characterized by a wide range of temperatures and chloride concentrations (Table 1). *Methanococcus* spp. requires much lower growth temperatures than *Methanothermococcus* spp., possibly (R command: ANOSIM; *p* = 0.018) explaining the shift in dominance from *Methanococcus* spp. to *Methanothermococcus* spp. between the cooler down-dip fields and the warmer up-dip fields (Whitman and Jeanthon, 2006, Figure 4). *Methanococcaceae* generate methane from either formate or CO_2_/H_2_ (Liu and Whitman, 2008), confirming previous conclusions that methanogenesis in this region is predominantly hydrogenotrophic (Warwick et al., 2008; McIntosh et al., 2010).

**Figure 4:**
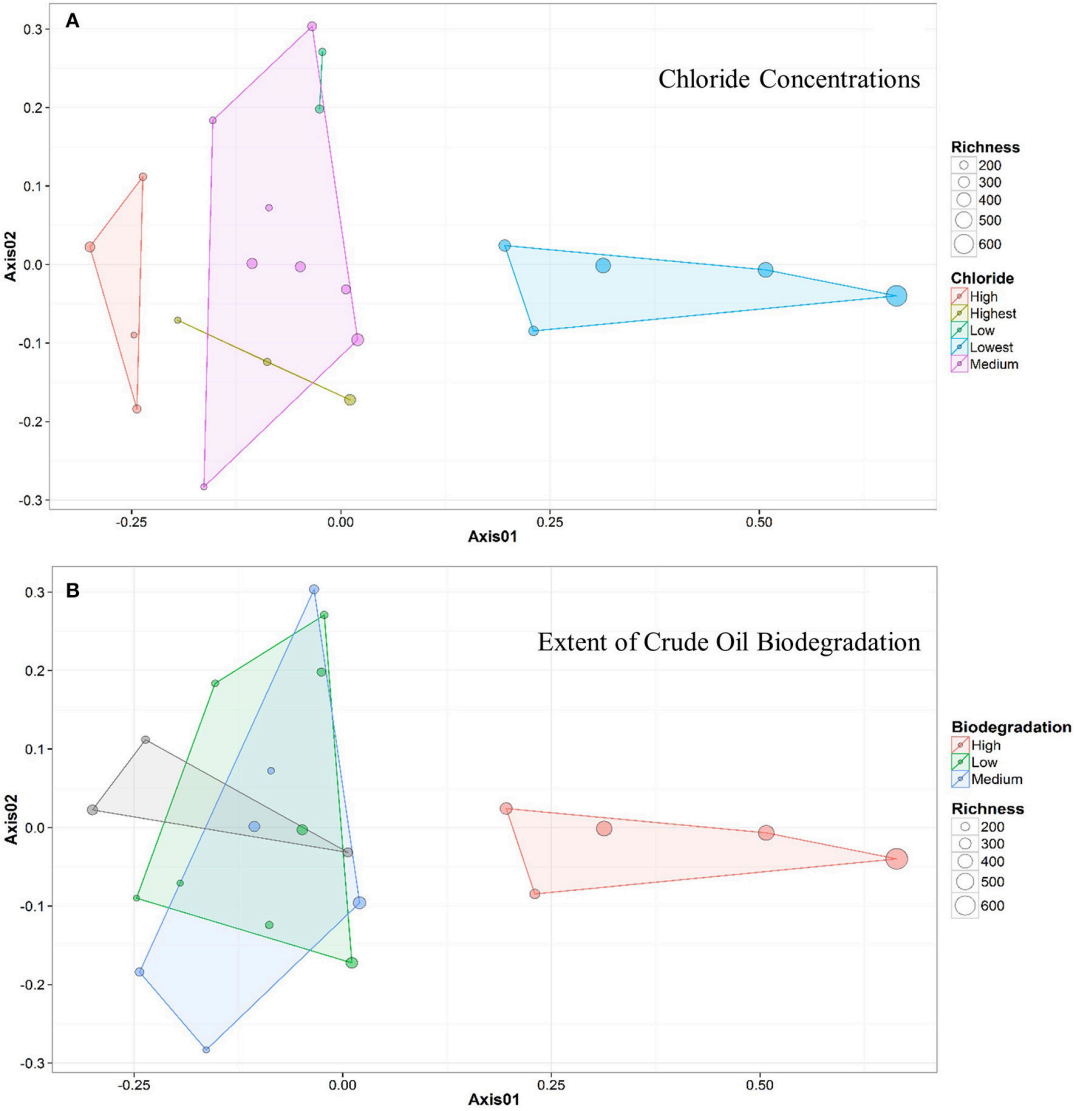
**NMDS ordination plots for wells clustered by (A) chloride concentration and (B) samples clustered by extent of crude oil biodegradation**. The distance between two points represents similarity between their microbial community structure, with greater distance indicating a greater difference in average microbial community composition. The colors of both the points and the associated polygons (or lines) correspond to the group the samples are clustered into (e.g., Medium vs. Low), and the size of the point corresponds to the sample richness. Both plots indicate that 5 samples (to the right) generally cluster away from the remainder of the samples (to the left).

The remaining two wells were dominated by either *Methanohalophilus halophilus* or *Methanolobus* spp. The dominance of *M. halophilus* in well O6 was surprising based on the preferred high salinity growth for the organism—1200 mM Cl^−^ (Kendall and Boone, 2006)—and also because well O6 was not the most saline well sampled. *Methanolobus* spp., which is present at the highest percent abundance in well NH2, is a methylotrophic methanogen that cannot use H_2_/CO_2_, acetate, or formate to grow (Kendall and Boone, 2006). Again, no preferential growth conditions for *Methanolobus* spp. would justify the presence of *Methanolobus* spp. in well NH2 over other wells across the transect.

Bray-Curtis distances were calculated (R commands: decostand; vegdist) to assess similarity in community composition across the sampled wells (Figure 2). The 5 up-dip, low-salinity, highly biodegraded wells (located in the CR, S, and TU fields) clustered (according to microbial community composition only) away from the remaining 17 wells (R command: hclust), which spanned all ranks of methanogenesis (high, medium, and low). NMDS ordination (also using Bray-Curtis distance) also visualized (R command: metaMDS) that the 5 up-dip shallow wells (CR, S, and TU) clustered together. However, they only clustered together when the data were analyzed against 3 of the 11 different environmental parameters, binned chloride concentration, extent of crude oil biodegradation, and oil field/depth (Figure 4), indicating that these parameters may be driving microbial community compositional differences across the transect. The Kruskal-Wallis test (R command: kruskal.test) found that microbial community richness (i.e., number of OTUs present in each sample) was significantly different (*p* < 0.05; Table S2) between groups of samples binned by spatial location within the reservoir (i.e., oil field/depth), pH, and extent of crude oil biodegradation. The number of identified OTUs in the shallow, high pH, highly biodegraded samples was generally greater than those identified in the deeper, lower pH, and low biodegradation wells.

Spatial location within the reservoir, chloride concentrations, and extent of crude oil biodegradation all gave significant *p*-values (≤ 0.001), and statistical *R*-values greater than or equal to 0.5 (Table S2) when applying the ANOSIM test (R command: anosim). This means that for those three categorizes, there is a statistical difference between the average community compositions of the binned wells analyzed for each of the categories (i.e., the wells binned by high chloride concentrations have an average microbial community composition that is different than those binned by low chloride concentrations). The ANOSIM test was repeated for just the bacteria identified in each well and just the Archaea identified in each well. When just analyzing the bacteria found in each well, the ANOSIM test only resulted in one significant grouping—by chloride concentration (Table S2). When just analyzing the archaea, the ANOSIM test identified two different significant groupings—extent of crude oil biodegradation and alkalinity. This suggests that both the Archaea and bacteria found in each well may be driving the amount of crude oil biodegradation found in each well, that crude oil biodegradation was accompanied by microbial community growth, and/or that the Archaea may impact alkalinity concentrations, independent of the bacteria.

### Microbial community differences between the up-dip and down-dip samples

Due to the sample clustering visualized using NMDS (e.g., the 5 up-dip wells clustered away from the 17 other wells), these 5 samples were removed from the statistical analysis. The ANOSIM test was performed again to determine if those 5 up-dip samples were driving the microbial community differences observed across the transect, or if there were still microbial community structure differences in the remaining 17 wells (i.e., is the variability identified exclusive to the 5 up-dip samples?). When removing those 5 samples, hydrogeochemical differences were still abundant across the remaining 17 samples; the remaining 17 wells span 3 different oil fields, all extents of methanogenesis (high, medium, and low), produced fluids with temperature values from 24.4 to 48.5°C, pH values between 6.7 and 7.4, δ^13^C-DIC values from +0.2 to +24.1%0, alkalinity values between 4.7 and 56.4 meq/kg, and chloride concentrations between 1154 and 2056 mM (Table 1). After removing those 5 samples and re-running the ANOSIM test, no environmental parameters were associated with significantly different microbial structures (*p* > 0.05; Table S2). This indicates that the major microbial community variations are found in the 5 up-dip samples, and that the remaining 17 samples have statistically similar microbial communities when binned by environmental parameters.

When analyzing the average order-level taxonomy of the two groups of samples (5 up-dip wells vs. the 17 down-dip wells), there were some major differences in microbial structure. Thirty-five different orders were identified at, on average, greater than 0.5% abundance in either of the two groups of samples. *Methanococcales* was the dominant order in both groups of samples; however, *Methanococcus* spp. dominated the up-dip wells while *Methanothermococcus* spp. dominated the down-dip wells (Section Differences in Microbial Community Composition across the Transect). No other identified orders were found at greater than 10% abundance in both groups. Some specific orders were found at a much higher percent abundance (or are only present) in the up-dip wells, and vice-versa. Most notably, greater than 90% of the identified sequences of *Cloacamonales, Bacteroidales, Chromatiales, Desulfobacterales, Desulfovibrionales, Desulfuromonadales, Methanobacteriales*, OPB11, PL-11B10, *Spirochaetales*, and YLA114 were found in the up-dip oil fields, while greater than 90% of the identified sequences of *Halanaerobiales, Oceanospirillales*, and *Synergistales* were found in the down-dip wells.

All of the orders containing sulfate reducing bacteria (SRB) were found in much higher abundance in the up-dip, low salinity, highly biodegraded, non-methanogenic wells. These include *Desulfobacterales, Desulfovibrionales, Desulfuromonadales, Chromatiales*, and *Syntrophobacterales*. This would support a hypothesis made in Shelton et al. (2016) that these up-dip highly biodegraded wells are minimally methanogenic due to sulfate reduction being the primary crude oil biodegradation pathway in these wells. The presence of SRB could limit methane production in these 5 up-dip wells by creating an increase in electron donor consumption by sulfate-utilizers, relative to methanogens, suggesting methanogens are being out-competed for electron donors (e.g., Bethke et al., 2011) and thus, limited in function. *Bacteroidetes*, identified in wells for this study, have been identified in other crude oil and methane producing reservoirs, while novel microorganisms similar to *Bacteroidales* have also been identified in biodegraded reservoirs (Wang et al., 2011; Purwasena et al., 2014), suggesting that *Bacteroidales* may be able to degrade crude oil. One Family of *Syntrophobacterales, Syntrophorhabdaceae*, have been shown to degrade organic compounds to acetate, in a syntrophic relationship with an H_2_ utilizer (Qiu et al., 2008; Kuever, 2014). Another species within the Order of *Syntrophobacterales, Desulfobacca acetoxidans* (*Desulfobacca* spp. was identified in this study), was identified as a sulfate reducer that competes for acetate with methanogens in sludge (Göker et al., 2011). *Methanobacteriales* is a methanogenic Order of bacteria within the Archaea that are typically identified as CO_2_/H_2_ utilizers (Bonin and Boone, 2006; Whitman et al., 2006), and have also been identified in methanogenic petroleum reservoirs or enrichment cultures (e.g., Wang et al., 2011; Mayumi et al., 2013).

A recent study by Hu et al. (2016) discussed the role of candidate Phyla in the biodegradation of crude oil, finding that the Phylum OP9 (termed *Atribacteria*) dominated samples that exhibited the most crude oil biodegradation (while candidate Phyla in the other, less-biodegraded samples ranged from 0 to 0.4% abundance in each sample). Carr et al. (2015) also found *Atribacteria* (consisting of both OP9 and JS1 candidate Phyla) in a methane-rich environment and suggested that it produced methanogenic substrates such as acetate and CO_2._ Although, the microbial structure of the highly biodegraded samples in this study do include many candidate Phyla, OP9 is not identified; OP9 is identified in the methanogenic, mid to low biodegradation samples. The absence of *Atribacteria* in the highly biodegraded samples may impact syntrophy between fermentative bacteria and methanogens in these wells, which could be limiting methane production.

OPB11, PL-11B10, *Cloacamonales*, and YLA114 are all found, on average, at greater than 0.6% abundance in the up-dip, highly biodegraded samples, and found at less than 0.05% abundance in the down-dip, less biodegraded samples. This abundance, combined with anosim test results comparing these four orders to degree of biodegradation (*p* = 0.002) suggests that OPB11, PL-11B10, *Cloacamonales*, and YLA114 may serve an important role in degrading hydrocarbons. OPB11, a candidate order of the Class *Anaerolineae*, may degrade organic matter: *Anaerolineae* are hypothesized to consume hydrocarbon intermediates (Kleinsteuber et al., 2012), while members of the Class *Anaerolineae* have been identified as organic matter degraders (Hug et al., 2013). PL-11B10, an order of the Phylum *Spirochaetes*, may also degrade organic matter, as *Spirochaetes* was identified as a dominant Phylum in production waters from a biodegraded, low-salinity petroleum reservoir (Grabowski et al., 2005). The Phylum WWE1, which includes the Order *Cloacamonales*, was recently identified in fluids collected from coalbed methane wells (Kirk et al., 2015), and was previously suggested to be a cellulose degrader found in anaerobic sludge digesters (Limam et al., 2014), likely indicating a hydrocarbon degrading role for *Cloacamonales*. The Order YLA114 is found in the new Phylum *Parvarchaeota*; little information exists on the presence of *Parvarchaeota* in methanogenic, biodegraded crude oil reservoirs.

### Factors driving microbial and metabolic differences across the transect

These results indicate that the extent of methanogenesis, which varies widely across the sampled transect, is not impacted by either the bacterial or archaeal communities present in each well. This may suggest that although “who's there” (i.e., the microbial communities identified at the time of sampling) may not have influenced the amount of methanogenesis, that other factors (possibly salinity or actual abundances of archaea and bacteria) may control whether or not certain communities are active and will generate methane. It is also possible that the microbial community has changed dramatically over time, and that the microbes present during the onset of crude oil biodegradation and methanogenesis may not have been captured by our sampling efforts. However, the extent of crude oil biodegradation may indeed be impacted by the methanogens present, as indicated by the results of the ANOSIM test. This evidence would further confirm that crude oil biodegradation and methanogenesis are linked in subsurface petroleum reservoirs (e.g., Jones et al., 2008; Gieg et al., 2010; Shelton et al., 2016), and that Archaea may play a major role in degrading the hydrocarbons, not just in generating methane.

Current research also suggests that there may not be major differences in the microbial communities present in biodegraded vs. non-biodegraded reservoirs (Sette et al., 2007; Oliveira et al., 2008), and that the major difference may be the relative abundance of the populations present (i.e., species evenness). This conclusion is not in agreement with the findings from this study. Bacterial richness is much higher on average in the oil fields exhibiting higher amounts of crude oil biodegradation (up-dip fields) than ones exhibiting minimal crude oil alteration (down-dip fields). The average Pielou evenness value (Table 2) is also similar between the 5 up-dip (0.81) and the 17 down-dip (0.80) samples, indicating similar evenness for the highly biodegraded samples compared to the less biodegraded samples. Furthermore, there are significantly different microbial community structures between the highly biodegraded and moderately/minimally biodegraded areas of the reservoir, indicating different microbial communities (and differences in their abundance) surrounding these wells. However, this could be due to the hydrogeochemistry of the reservoir, as the more degraded fields are associated with lower temperature and more dilute fluids than the deeper oil fields.

Salinity has been shown to be a major driver of microbial community presence and metabolism, affecting both crude oil biodegradation and microbial methanogenesis (e.g., Potter et al., 2009; Oren, 2011; Head et al., 2014). Shelton et al. (2016) suggested that chloride concentrations were driving differences in the amount of crude oil biodegradation across the transect sampled in this study. This study also provides evidence to support salinity as a driver of microbial community differences (as shown by a significant ANOSIM *p*-value). There are two major changes in salinity concentrations across the transect: between the shallow wells and the mid-depth wells, and between the mid-depth well and the deep wells.

However, it is important to note that major changes in salinity, spatial location, and extent of crude oil biodegradation exist when comparing the 17 down-dip wells, even though there appears to be no major microbial structure differences across these 17 wells. The 17 down-dip wells span three different oil fields and 921 m of depth, produce formation water with chloride concentrations between 1154 and 2056 mM (Table 1), and produce crude oil that is either moderately or minimally biodegraded. Therefore, it is difficult to say that, “salinity is driving microbial community differences across the transect,” because there are major salinity differences present down-dip, with fairly similar average microbial communities present along that gradient.

It is more prudent to hypothesize that a salinity threshold may exist between 776 and 1154 mM that impacted the microbial community structure in this formation. Crude oil biodegradation may be limited above this hypothesized threshold, because there is a major difference in crude oil biodegradation between wells producing water with these different chloride concentrations. It is difficult to determine if methanogenesis itself is limited at lower salinity values, or if methanogens have been (or are being) outcompeted by SRB around these wells locations. Therefore, microbial function in these 5 up-dip wells may not be limited by salinity specifically, but rather by the lack of sulfate present to support the SRB identified in the 5 up-dip wells.

High concentrations of salinity may not impact microbial community structure, as indicated by the results of this study (e.g., the 17 down-dip wells), but merely microbial function (i.e., metabolism and roles). Both crude oil biodegradation and microbial methanogenesis are limited in the most-saline field sampled, the field with wells producing formation water with chloride concentrations >2000 mM. As the microbial community structure in the highly-saline fields is statistically similar to the structure of the centrally located methanogenic field, it is fair to assume that salinity is limiting the lack of function in these down-dip fields (i.e., limiting methanogenesis and/or crude oil biodegradation).

## Conclusions

Archaea, on average, dominated the microbial community composition in each sampled location, while a methanogen, regardless of the percent Archaea, was the dominating microbial species in each sampled well location. Statistical analyses performed on the total community structure suggested that chloride concentrations, spatial location within the Gulf Coast Basin (i.e., well depth and surficial location), and the extent of biodegradation have the most influence on the microbial community structure across the transect. However, salinity and spatial location within the reservoir are co-linear, and are associated with one another. The statistical variability between microbial structure and environmental parameters is due mostly to processes occurring in 5 specific well locations, where the shallowest wells produce fluids with the lowest chloride concentrations and highest extent of crude oil biodegradation. After removing the data from these 5 wells from the statistical analyses, no significant differences in microbial community structure exist when the remaining 17 wells are binned by environmental parameters, even though environmental gradients still exist (i.e., changes in salinity, temperature, and pH are prevalent). Therefore, we hypothesize that either (1) a chloride threshold (between approximately 800–1000 mM) exists, resulting in a dramatic shift in the microbial community structure between the 5 up-dip well locations and the 17 down-dip well locations; and/or (2) the major difference between these two groups of sample locations is due to differences in crude oil biodegradation, likely stemming from variations in metabolism (sulfate reduction vs. methanogenesis). Furthermore, this salinity threshold may also be driving the high amounts of crude oil biodegradation observed in the low salinity, up-dip well locations, leading to the conclusion that salinity is the major driver in controlling microbiology across the transect. Salinity does not appear to impact microbial community structure beyond this threshold, as indicated by the lack of major difference in microbial community structure in the down-dip wells. Variations in methanogenesis across the transect are not linked to statistically different microbial communities, and therefore, as hypothesized in Shelton et al. (2016), are likely due to hydrogeochemistry and oil quality.

This research has implications for enhancement of natural gas production from crude oil biodegradation. Although, methanogens were abundant, represented by low species diversity, and found in every well sampled for this study regardless of the apparent extent of methanogenesis, extent of methanogenesis still varies across the transect. This implies that engineering reservoirs to favor specific methanogen populations may not be useful when trying to accelerate methanogenic crude oil biodegradation, because hydrogeochemical conditions of the reservoir may be affecting the methanogens' ability to convert organic substrates to natural gas. Crude oil biodegradation is likely limited by either salinity or by microbial ecology, as both change drastically between the 5 up-dip, low-salinity, highly biodegraded samples and the remaining 17 down-dip, higher salinity, low biodegradation samples. Methanogenesis seems to be more halotolerant than crude oil biodegradation, however the hypothesized switch from sulfate reduction to methanogenesis in the shallow, up-dip fields would need to be further investigated to determine if methanogenesis will occur in this low-salinity, low oil quality environment.

## Author contributions

JLS helped devise the research plan, performed field work, analyzed samples and resulting data, and wrote and edited the manuscript. DA assisted in devising the research plan, analyzing the samples and resulting data, and edited the manuscript. JCM assisted in devising the research plan, analyzing the data, and edited the manuscript. NF assisted in analyzing the samples and the resulting data, as well as in editing the manuscript. JRS assisted in devising the research plan, analyzing the data, and in editing the manuscript. PW assisted in devising the research plan and in editing the manuscript. JEM assisted in devising the research plan, analyzing the data, and in editing the manuscript.

### Conflict of interest statement

The authors declare that the research was conducted in the absence of any commercial or financial relationships that could be construed as a potential conflict of interest.
