# Supplementary figures and images for: Environmental Drivers of Differences in Microbial Community Structure in Crude Oil Reservoirs across a Methanogenic Gradient
Source: Front Microbiol. 2016 Sep 28;7:1535. doi: 10.3389/fmicb.2016.01535 (PMC5039232; doi:10.3389/fmicb.2016.01535)

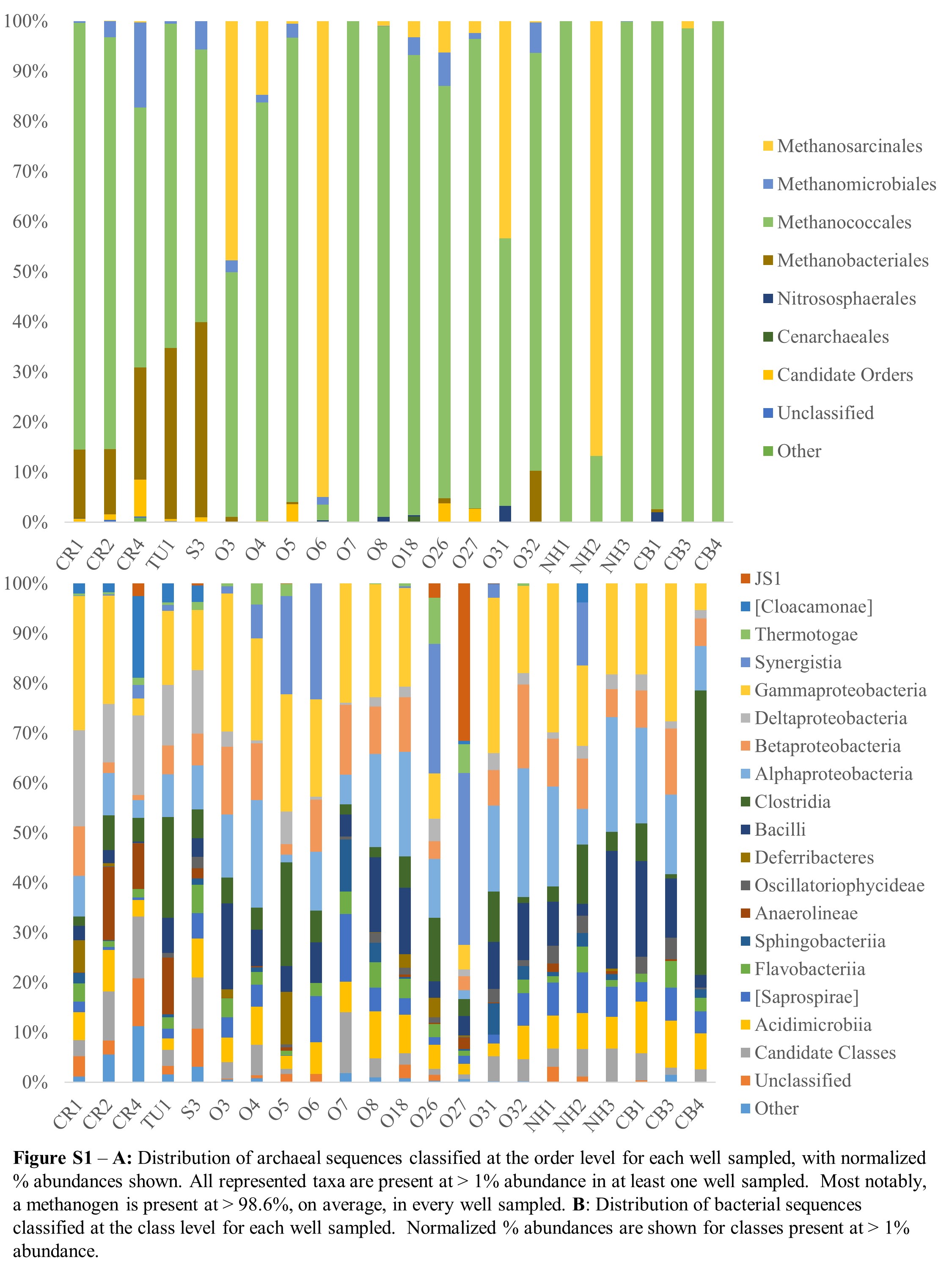

Supplement: Supplementary file 3 [file Image1.JPEG]
